# Integrative Transcriptome Profiling of Cognitive Aging and Its Preservation through Ser/Thr Protein Phosphatase Regulation

**DOI:** 10.1371/journal.pone.0130891

**Published:** 2015-06-23

**Authors:** C. Sehwan Park, Amandine Valomon, Hans Welzl

**Affiliations:** 1 Brain Research Institute, Medical Faculty of the University of Zürich and Department of Health Science and Technology ETH Zürich, Zürich, Switzerland; 2 Institute of Pharmacology and Toxicology, Human Sleep Psychopharmacology, University of Zürich, Zürich, Switzerland; 3 Institute of Anatomy, Neuroanatomy and Behavior, University of Zürich, Zürich, Switzerland; The Florey Institute of Neuroscience and Mental Health, AUSTRALIA

## Abstract

Environmental enrichment has been reported to delay or restore age-related cognitive deficits, however, a mechanism to account for the cause and progression of normal cognitive decline and its preservation by environmental enrichment is lacking. Using genome-wide SAGE-Seq, we provide a global assessment of differentially expressed genes altered with age and environmental enrichment in the hippocampus. Qualitative and quantitative proteomics in naïve young and aged mice was used to further identify phosphorylated proteins differentially expressed with age. We found that increased expression of endogenous protein phosphatase-1 inhibitors in aged mice may be characteristic of long-term environmental enrichment and improved cognitive status. As such, hippocampus-dependent performances in spatial, recognition, and associative memories, which are sensitive to aging, were preserved by environmental enrichment and accompanied by decreased protein phosphatase activity. Age-associated phosphorylated proteins were also found to correspond to the functional categories of age-associated genes identified through transcriptome analysis. Together, this study provides a comprehensive map of the transcriptome and proteome in the aging brain, and elucidates endogenous protein phosphatase-1 inhibition as a potential means through which environmental enrichment may ameliorate age-related cognitive deficits.

## Introduction

Aging is associated with a deterioration of learning abilities and memory retention, which is often progressive and debilitating. Although usually persistent, age-related cognitive decline (ARCD) may be preventable or delayed and, in some conditions, cognitive functions can be partially or completely reinstated. In particular, environmental enrichment (EE) and physical exercise, separately or together, have been shown to promote neurogenesis [1], increase synaptic plasticity [2], and rescue cognitive deficits in aged mice [3–5], in mouse models of neurodegeneration [6,7], in Alzheimer’s and other neurological diseases [8–10].

The underlying mechanism(s) leading to progressive ARCD remains unknown but may involve protein phosphatases (PPs), in particular protein phosphatase-1 (PP1) and calcineurin, because they are strictly modulated by intracellular calcium and are negative regulators of NMDA receptor signaling, synaptic plasticity, learning and memory [11–15]. Furthermore, in aged rodents, dysregulation of calcium homeostasis and PP activity has been associated with cognitive deficits [16–19] and Alzheimer’s disease [12,20–22].

In addition to dysregulated cellular signaling pathways, gene expression profiles in the brain have been found to be altered in aging [23–25] and Alzheimer’s disease [26–28]. In the hippocampus, activity-dependent gene transcription is rapidly induced and protein synthesis is required for the formation of long-term memory [29–31]. However, in ARCD, there is a general decrease in transcriptional activity of various gene networks [24,32,33], as well as a decrease in the expression of immediate-early genes [23,34,35]. These studies suggest that the underlying cause of ARCD associated with cellular senescence and dysregulated biological processes in the brain may be a consequence of altered transcriptional programs. As of yet, however, few genes have been identified to target ARCD, and no consensus mechanism has been attributed to ARCD or the beneficial effects of EE to restore cognitive functions.

Here we use high-resolution serial analysis of gene expression followed by deep sequencing (SAGE-Seq) in combination with quantitative isobaric tag for relative and absolute quantitation (iTRAQ) proteomics to profile the hippocampal transcriptomes of aged and young mice. Our findings suggest that regulation of PP1 activity through the endogenous expression of PP1 inhibitors may underlie EE-mediated amelioration of ARCD and may provide a potential target for intervention.

## Materials and Methods

### Animals and ethics

Middle-aged (15–17 months at the beginning of the experiments) and young adult (5–6 months) C57Bl/6J male mice were used for the following behavioral experiments. Mice were either housed in standard cages (SH) (S1A Fig) or housed in environmentally enriched cages (EE) (S1B Fig). SH mice were housed in a maximum of four animals to a cage in clear, polycarbonate standard Aero cages measuring 391 x 199 x 160 mm, with a floor area of 778 cm^2^ (S1A Fig), while EE mice were housed in a maximum of six animals to a cage in clear, polycarbonate type 2000P cages measuring 610 x 435 x 215 mm, with a floor area of 2654 cm^2^ (Tecniplast) (S1B Fig). After initial behavioral assessment, mice were housed between 6 and 11 weeks in EE or SH environments respective to their experimental condition.

EE cages consisted of a rearrangeable set of tunnels and houses, two running wheels per cage for physical activity, climbing ladders and jungle gyms, and several sets of novel objects, including plastic balls, textured rubber balls, balls with bells, wooden sticks, pipe rings, and corn cobs which were introduced weekly and arranged randomly (S1C and S1D Fig). All animals were provided with access to food and water, *ad libitum*, under a reverse 12 hour light/dark cycle (dark 8 am– 8 pm). All animals were maintained in accordance with the Federation of Swiss Cantonal Veterinary Office and European Community Council Directive (86/609/EEC) guidelines. Animals from the same cohort were sacrificed on the same day by rapid cervical dislocation. This research was approved by the Swiss Cantonal Veterinary Office under Licenses 150/06 and 105/2008.

### Open field and 2-context contextual fear conditioning

Mice were individually recorded for 6 minutes in a 1 x 1 m grey plastic container with 40 cm high walls using the Viewpoint video tracking system (Viewpoint Life Sciences, Lyon, France). Time spent in the center of the box, defined as the distance 20 cm from each wall, was used as a general measure of exploration and anxiety-related behavior.

Contextual fear conditioning was performed using a TSE Fear Conditioning System and was performed as previously described [36,37]. Mice were trained and tested before and after EE or SH using two different contexts in the conditioning chamber. Two different contexts were used to train and test the mice twice and prevent association to the previous context. Context A consisted of the default clear plastic container and grid floor from TSE (S2A Fig). Context B consisted of a black plastic container and custom-made floor made of 1.25 cm metal strips with regularly spaced drilled holes measuring 0.5 cm in diameter (S2B Fig). Mice were trained and tested in either context A or context B and were counterbalanced for the two contexts. After 2 months of respective housing, mice that were trained and tested in context A were subsequently trained and tested in context B, and those in B in A.

Training consisted of 3 min exposure to the context followed by a brief electric shock (0.7 mA for 1s) and left in the context for an additional 3 min. The first 3 min of exposure to the context in the training session was used as baseline. Mice were then tested 24 hours later without a shock and time spent freezing was recorded in 3 min re-exposure to the contexts. Baseline freezing was subtracted from freezing levels in the test session to normalize age differences. A cohort of 18 aged and 16 young mice were used for this study. Mice were habituated (Hab) for 1 week prior to behavioral testing and sacrificed (End) two weeks after the last test.

### 2-context Morris water maze

The Morris water maze task (MWM1) was performed as previously described [14,15,38], in a pool measuring 1.5 m in diameter. Two days of pre-training to a visible platform, was followed by 10 days of training (acquisition) to a hidden, circular plastic platform in the target quadrant, and a probe trial on day 11. Training consisted of 3 trials per day with an inter-trial-interval (ITI) of 60 min, with each start position at one of the three non-target quadrants. Latency was measured as the time needed to find the hidden platform. The probe trial involved a 60 s search of the maze with the platform removed and measured as the percent time spent in each quadrant. The second Morris water maze task (MWM2), after two months of respective housing, was performed in a different facility with different external cues, different lighting, a square metal platform, a 10 min ITI, and a pool measuring 1.2 m in diameter. No pre-training was performed in MWM2 and acquisition training was reduced to 9 days. Behavior was assessed using an automated video tracking system EthoVision (Noldus). A cohort of 22 aged and 22 young mice were used for this study. Mice were habituated (Hab) for 1 week prior to behavioral testing and sacrificed (End) five weeks after the last test, and hippocampal tissue was processed for SAGE-seq.

### Displaced and novel object recognition (DNOR)

The object recognition task was performed as previously described with the addition of a displaced object test [14,15,38]. Briefly, training involved exposing mice to an arena containing three unfamiliar objects for 5 sessions of 5 min with an inter-trial interval (ITI) of 5 min between each session. Objects were chosen based on equal preference. The memory test 24 hours after training was comprised of a 5 min session with one object displaced (Displaced), followed by a 5 min interval, then a 5 min session with one of the previous objects replaced (Novel). Behavior was assessed using an automated visual tracking system (Viewpoint) and discrimination ratio was calculated as time spent with the target object (novel or displaced) divided by the total time spent with all objects. A cohort of 17 aged and 17 young mice were used for this study. Mice were habituated (Hab) for 1 week prior to behavioral testing and sacrificed (End) 1 day after testing, and hippocampal tissue was processed for the protein phosphatase assay.

### Protein phosphatase activity assay

Protein phosphatase assays were performed using the Calcineurin Cellular Activity Assay Kit (Enzo Life Sciences) as described with modifications. We chose 6 mice randomly from each group of the DNOR task and ran one group against another–EA versus SA and EY versus SY–simultaneously on the same 96-well plate to reduce experimental variability. Briefly, hippocampi were dissected and homogenized in 250 μl of lysis buffer with protease inhibitors and centrifuged at 14,000 g for 30 min. The supernatant containing cytoplasmic fraction was desalted using inorganic PiBind resin (Innova Biosciences). Phosphatase activity was determined by incubating 0.5 μg of sample with 0.75 mM of RII phosphopeptide substrate with either EGTA or with 5nM tautomycin (TM) for 60 min at 25C. Release of free phosphates during the reaction was measured with BIOMOL Green reagent at 620 nm with the background subtracted. PP1/PP2A and calcineurin activity was calculated by the amount of phosphate released in the presence of EGTA or tautomycin, respectively.

### Statistical analysis

Statistical analysis for behavioral experiments and the protein phosphatase assay was performed using GraphPad Prism 6. Two-way, repeated measures analyses of variance (ANOVA) corrected for multiple comparisons, one-way ANOVA followed by Newman-Keuls post hoc test or two-tailed Student’s *t*-test were used where appropriate. Statistical significance was set at *p < 0.05, ••p < 0.01, and •••p < 0.001. All data are shown as mean ± s.e.m.

### Sample and SAGE-Seq library preparation

Total RNA was extracted from one hippocampus from each animal using the TRIzol procedure (Invitrogen) and processed in parallel. RNA quality was assessed using an Agilent Bioanalyser 2100 and quantified using a Qubit 2.0 Fluorometer (Invitrogen). All RNA samples had an RNA Integrity Number score greater than 8, with an average of 8.43. SAGE tag library preparation was performed according to the SOLiD SAGE kit with Barcoding Adaptor Module (Invitrogen). Approximately 3 μg of RNA was used to isolate polyadenylated RNA using poly-dT Dynabeads and converted to cDNA using Superscript III Reverse Transcriptase. Samples were digested with sequence-specific Endonuclease *Nla* III restriction enzyme and ligated to barcoded adaptor A. The fragments were then released from the Oligo (dT) EcoP magnetic beads using *Eco*P15I restriction enzyme. The resulting fragments were then ligated to adapter B which contains the P1 sequence. 130 bp ligation products were PCR-amplified and purified using PureLink PCR Micro Kit (Invitrogen). Barcoded libraries were then quantified using a Qubit Fluorometer and pooled in equimolar volumes before emulsion PCR and sequenced on one full SOLiD4 slide (Applied Biosystems). From four groups belonging to the same MWM cohort–aged EE, aged SH, young EE, and young SH–three samples from each group were run in parallel as biological replicates.

### Sequence analysis, alignment and mapping

We sequenced approximately 11.6 Gb, equivalent to 387 million reads, from three biological replicates from each group, equivalent to approximately 4.6 complete mouse genomes. Color-space calling was performed using Bioscope version 1.3.1 using default parameters from Applied Biosystems. Mapping was performed with the SOLID SAGE perl script version 1.10 available from Applied Biosystems. Reads were aligned to mouse genome assembly NCBI37/mm9 (July 2007) with an allowance of 1 mismatch and converted into the bam file format using a custom script. Comparative counts on 29,973 transcripts comprising 22,910 unique genes, were performed using the GLM method implemented in the R/Bioconductor statistical package, EdgeR (version 2.4.6), with p < 0.05 and adjusted for multiple comparisons [39]. Differential gene expression profiles were established with a false discovery rate (FDR) < 0.10. Differentially expressed significant genes were clustered in an unsupervised fashion using an exact test method at a significance threshold of p < 0.01. Two biological replicates per group were chosen for comparative analysis based on Mann-Whitney-Wilcoxon tests on transcripts with an FDR < 0.10 [40]. Mice are referred to as follows: Aged EE (EA) are enriched aged, Aged SH (SA) are standard-housed aged, Young EE (EY) are enriched young, and Young SH (SY) are standard-housed young. Sequence data from this study has been submitted to NCBI Gene Expression Omnibus database (http://www.ncbi.nlm.nih.gov/geo/) and assigned the identifier (accession no. GSE43718).

### Gene ontology and pathway analysis

Gene ontology analysis of differentially expressed transcripts at Benjamini and Hochberg-adjusted p < 0.01 was performed using the Database for Annotation, Visualization and Integrated Discovery (DAVID, v6.7; david.abcc.ncifcrf.gov). Annotated GO terms were classified into higher order ancestral GO terms based on the GO slim classification method and counting by accumulative/all occurrences in the GO Terms Classifications Counter (CateGOrizer; www.animalgenome.org/tools). Count threshold of 2 and EASE score of 0.1 was used, a more conservative test than Fisher’s Exact test. GO terms were classified into ten broad functional groups and represented as a percentage of a specific category over all categories identified. Web-based Gene Set Analysis Toolkit V2 (WebGestalt; bioinfo.vanderbilt.edu/webgestalt) was used for the analysis of functionally-enriched genes in the respective gene lists [41]. Genes were analyzed using a hypergeometric test with multiple adjustments using the method of Benjamini and Hochberg and organized into their respective classes and pathway associations based on the Kyoto Encyclopedia of Genes and Genomes (KEGG; www.genome.jp/kegg/kegg2.html).

### Sample preparation and iTRAQ labeling

Hippocampal nuclear extracts were prepared with the use of a Nuclei Pure Prep isolation kit (Sigma). Briefly, hippocampi were homogenized in 1 ml of lysis buffer containing 1 M DTT, 10% Triton X-100, protease inhibitor cocktail, and protein phosphatase inhibitor cocktails I and II (Sigma). 2 ml of 1.8 M sucrose was mixed into the homogenates and layered on top of 1 ml of a 1.8 M sucrose cushion. Samples were then centrifuged at 30,000 g for 45 min to pellet the nuclei. The supernatant was removed and the nuclei was resuspended in 300 μl of nuclei lysis buffer and sonicated for 2 min on ice. 1 μl of benzonase was added and incubated for 30 min at 37C. Samples were then centrifuged at 13,000 g for 10 min and the supernatant containing nuclear lysates was transferred.

100 μg of protein was used for iTRAQ experiments. Each sample was precipitated with ice-cold acetone, resuspended in iTRAQ dissolution buffer, and cysteine-blocked according to the manufacturer’s instrucitons (Applied Biosystems). Samples were then digested overnight with trypsin (Promega) at 37C (1:13 enzyme:substrate), differentially labeled with iTRAQ reagents, and combined as per protocol. The peptide samples were then separated by strong cationic exchange (SCX) chromatography and enriched using reversed-phase (RP) HPLC as described previously [42].

### LC-ESI-MS/MS analysis of phosphorylated peptides using Orbitrap

Samples were analysed on an LTQ-OrbitrapXL mass spectrometer fitted with an additional high-energy collision dissociation cell (ThermoFischer Scientific, Bremen, Germany). The LTQ-OrbitrapXL was operated in data-dependent acquisition mode in full scan in the Orbitrap and CID MS/MS scans in the linear ion trap. Settings were as follows: precursor mass range from 700 to 16,000 Da; minimum signal-to-noise ratio of 10; precursor M/Z tolerance of 1.2; and a signal peak window m/z of between 112.5 and 121.5. The automatic gain control was set to 1e5 for full FTMS and 1e4 for ion trap MS/MS scans and dynamic exclusion was enabled.

MS and MS/MS data were searched using Mascot version 2.2.04 in the European Bioinformatics Institute’s mouse protein database. Searches were performed with the following modifications: phosphorylation (STY, variable), iTRAQ (K and N-terminal, fixed), and methyl methanethiosulfonate (C, fixed) with a maximum of one missed cleavage. Monoisotopic masses of +1, +2, +3 charged peptides were searched with a peptide tolerance of 6 ppm and fragment mass tolerance cut-off of 0.5 Da. Significance threshold was set at 0.05 and ions score cut-off was set at 0.05. Analysis of Mascot searches were performed as previously described [42].

## Results

### Middle-aged mice have associative and spatial memory performance equivalent to that of young mice

**Fig 1:**
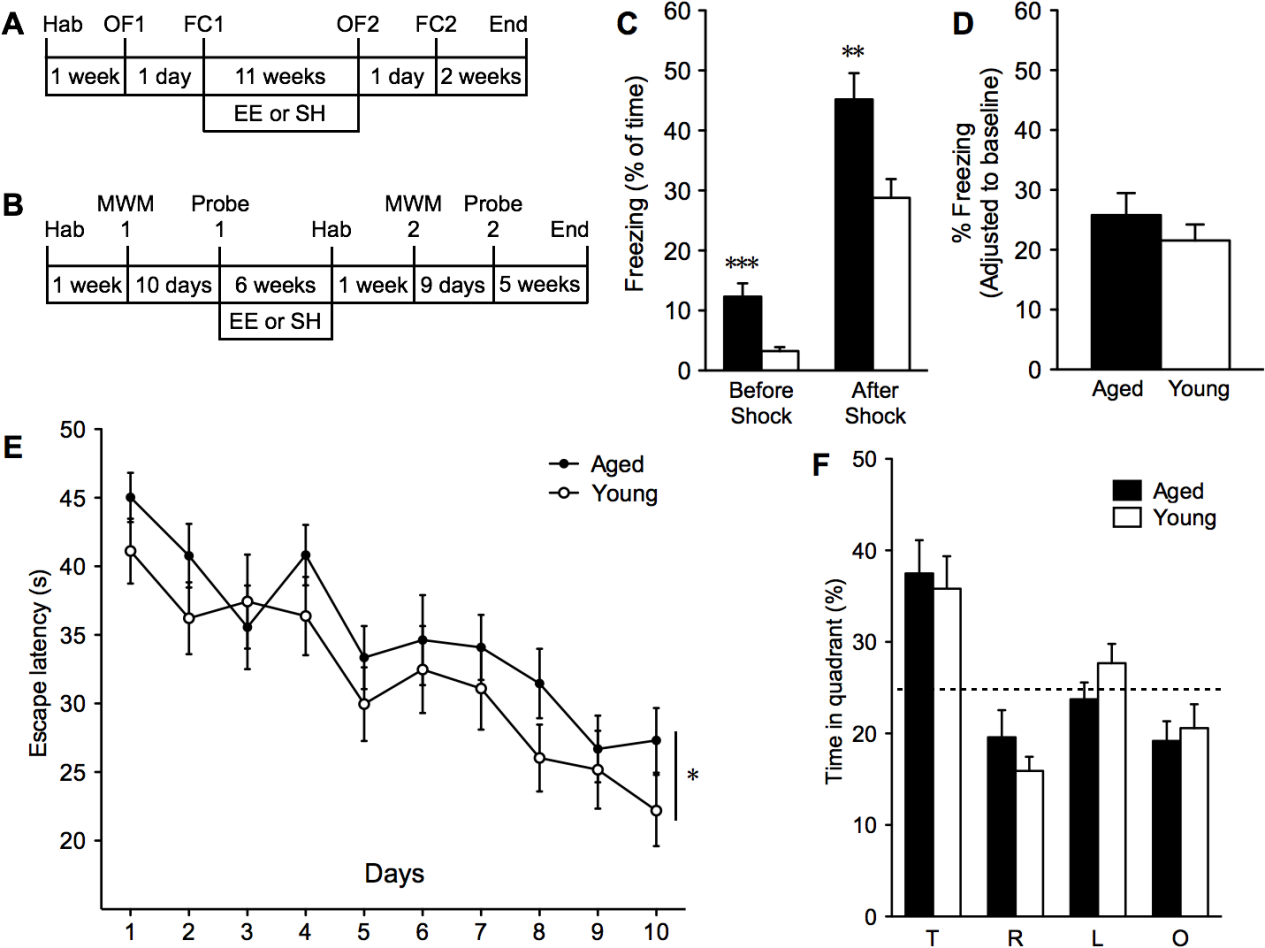
Assessment of middle-aged and young mice before separation into EE and SH. The schedule of contextual fear conditioning (**A**; 18 aged and 16 young mice) and Morris water maze tests (**B**; 22 aged and 21 young mice) before and after separation into respective housing conditions. Hab: habituation period, OF: Open field, FC: Fear conditioning, MWM: Morris water maze. (**C**) Fear conditioning in the first context and (**D**) accompanying memory test 24 hours later in the original context. (**E**) Escape latency to find the hidden platform in the Morris water maze test across 10 days of training and (**F**) probe trial on day 11 with the hidden platform removed (T, target; R, right; L, left; O, opposite). *p < 0.05, **p < 0.01, ***p < 0.001. Shown as mean ± s.e.m.

To assess the cognitive status of middle-aged (15–17 month) and young adult (5–6 month) mice prior to separation into EE and standard-housed (SH) cages, we first characterized a cohort of mice in open field and contextual fear conditioning tests (Fig 1A) and another cohort in the Morris water maze test (Fig 1B). Middle-aged mice (43.60 ± 1.44 g) weighed 43.7% more than young mice (30.34 ± 0.35 g; p < 0.001) at the onset of testing (S3A Fig). The open field test (OF1) showed no difference in the time spent in the center during 6 minutes of exposure, suggesting no changes in anxiety or exploratory behavior with age (S3B Fig). However, middle-aged mice had considerably higher freezing during 0 to 3 minutes of baseline exposure to the conditioning chamber (Before Shock; 12.31 ± 2.24% aged; 3.27 ± 0.65% young; p < 0.001) and 3 to 6 minutes following the shock (After Shock; 45.18 ± 4.38% aged; 28.78 ± 3.14% young; p < 0.01) compared to young mice in the contextual fear conditioning task (FC1; Fig 1C). After adjusting to baseline levels, we found no difference in freezing between middle-aged and young mice in the memory test 24 hours later (Fig 1D).

In a separate cohort of mice, we performed Morris water maze tests (MWM1). All mice learned the location of the platform with this protocol (ANOVA, effect of ‘days’, p < 0.001). During the acquisition phase, middle-aged mice required significantly longer times to find the hidden platform (ANOVA, effect of ‘group’, p < 0.05; Fig 1E) compared to young mice. However, the difference in acquisition time compared to young mice could be attributed to the slower swim speeds in middle-aged mice found in our study (ANOVA, effect of ‘group’, p < 0.001; S4A Fig) and others [24]. Middle-aged mice also had slower speeds in the probe trial on day 11 (p < 0.001; S4B Fig), but there was no difference in the average number of platform crossings (S4C Fig) and they retained the position of the hidden platform similar to young mice (Probe 1; Fig 1F). Our findings agree with previous reports showing minimal spatial memory deficits in middle-aged mice compared to young [43,44]. We then randomly separated the mice into EE or SH cages.

### Long-term environmental enrichment in middle-aged mice preserves contextual fear and spatial memory

Following several weeks of housing in either EE or SH cages, mice were re-assessed in their respective behavioral tests (Fig 1A and 1B). Pre-determined novel contexts were used for secondary behavioral evaluations in both fear conditioning and MWM tasks to avoid generalization to the original behavioral paradigms (see Materials and Methods). Middle-aged mice, now advanced in age and addressed hereafter as aged, reduced their weight differential (40.74 ± 1.51 g) and weighed only 24.7% more than young mice (32.65 ± 0.42 g; p < 0.001; S3C and S3D Fig). There was no effect of EE on the weight of young mice, but enriched aged mice weighed 16.1% less than standard-housed aged mice (Aged EE, 37.17 ± 0.91 g; Aged SH, 44.31 ± 2.39 g; p < 0.001; S3E Fig). Aged EE mice now reached a weight indistinguishable from that of standard-housed young mice (Young SH, 33.54 ± 0.58 g), but not that of enriched young (Young EE, 31.68 ± 0.46 g; S3E Fig). The effect of long-term EE and SH in a different cohort of mice show nearly identical changes in weight (S5D–S5G Fig).

**Fig 2:**
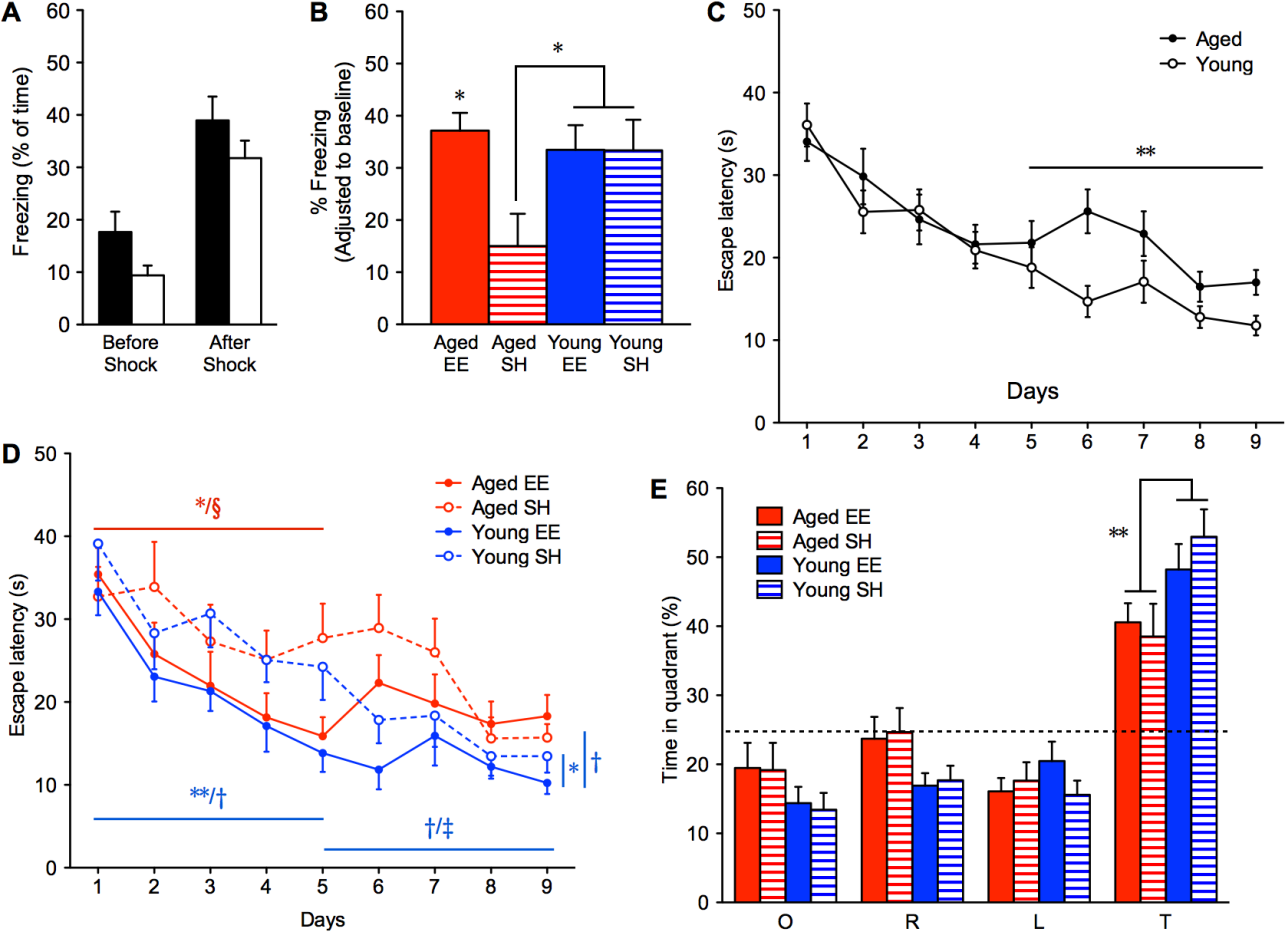
Assessment of aged and young mice after several weeks of undisturbed housing in EE or SH. (**A**) Fear conditioning in a novel context (FC2) and (**B**) memory test 24 hours later in the same context (n = 8, 6, 7, 7 for Aged EE (solid red), Aged SH (red stripes), Young EE (solid blue), Young SH (blue stripes), respectively; *F*_3,24_ = 3.646). Escape latency to a hidden platform in an alternate Morris water maze and room (MWM2), different than that of MWM1, (**C**) by age and (**D**) separated by age and housing conditions (n = 11, 11, 11, 10 for Aged EE (red line), Aged SH (red dashes), Young EE (blue line), Young SH (blue dashes), respectively; days 1–5, *F*_3,39_ = 5.524, p < 0.01; days 6–9, *F*_3,39_ = 4.307, p < 0.05; days 1–9, *F*_3,39_ = 4.652, p < 0.01; *p values shown with respect to aged (red) and young (blue); ^§^ *p* < 0.05 (Aged EE to Young SH); ^†^ *p* < 0.01 (Young EE to Aged SH); ^‡^ *p* < 0.05 (Young EE to Aged EE). Probe trial on day 10 with the hidden platform removed, (**E**) separated by age and housing conditions (O, opposite; R, right; L, left; T, target). *p < 0.05, **p < 0.01, ***p < 0.001. Shown as mean ± s.e.m.

There was no difference in time spent in center in the open field test following several weeks of housing (OF2), suggesting no improvement or impairment in exploratory behavior or anxiety with age and housing (S3F Fig). In the re-assessment of the fear conditioning task (FC2), baseline freezing (Before Shock; 0 to 3 minutes) remained similar to FC1 following the unconditioned stimulus (After Shock; 3 to 6 minutes; Fig 2A). Importantly, the mice did not associate the novel context with the original context and both young and aged mice could be fear conditioned a second time. After adjusting to baseline freezing levels, aged EE mice (37.13 ± 3.38%) exhibited freezing which was significantly higher than aged SH (15.03 ± 6.18%; ANOVA, effect of ‘group’, p < 0.05) and was equivalent to levels in young mice (Fig 2B).

To re-evaluate spatial memory performance following several weeks of housing, mice were re-trained and tested in a second novel Morris water maze (MWM2). Aged mice acquired the target in the same amount of time as young mice in the first five days (ANOVA, effect of ‘days’, p < 0.001), however, young mice continued to improve with additional training (days 5 to 9; ANOVA, effect of ‘group’, p < 0.01; Fig 2C). Aged EE mice also had lower escape latencies in the first five days than aged SH mice (*p < 0.05; Fig 2D). In fact, aged EE mice performed equivalent to young EE mice and even better than young SH mice across days 1 to 5 (^§^p < 0.05), but across days 6 to 9, there was no difference with young SH and they fared worse than young EE mice (^‡^p < 0.05; Fig 2D). Although swim speed was a factor between aged and young (ANOVA, effect of ‘group’, p < 0.001; S4D and S4E Fig), aged EE mice acquired the platform as well as young EE mice throughout training despite the handicap. Interestingly, young EE mice had significantly faster acquisition times across days 1 to 5 (**p < 0.01) and overall in the 9 days (*p < 0.05) than young SH mice, whose learning was more linear (Fig 2D).

In the probe trial, both young and aged mice spent more time in the target quadrant than by chance alone (ANOVA, effect of ‘quadrant’, p < 0.001), however, young mice spent significantly longer time (p < 0.01; Fig 2E) and had a higher number of average platform crossings in the target quadrant than aged mice (p < 0.05; S4F Fig). There was no difference in time spent in the target quadrant and the average number of platform crossings when separated by housing (ANOVA, effect of ‘group’, Fig 2E and S4G Fig).

### Environmental enrichment preserves recognition memory in middle-aged mice

**Fig 3:**
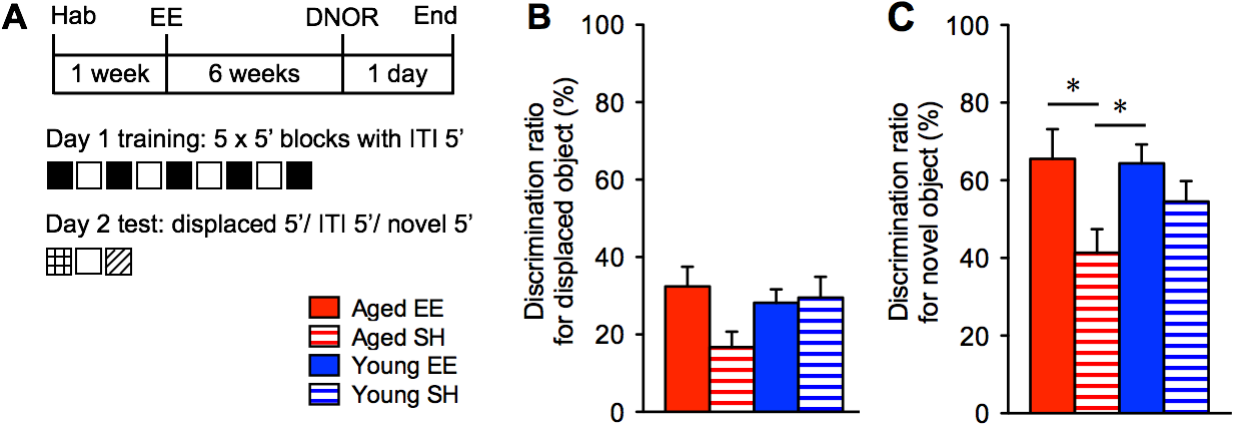
Recognition memory in aged and young mice following respective housing conditions. (**A**) Experimental design of the displaced and novel object recognition memory (DNOR) tasks over two days in 5 min blocks with 5 min ITI (n = 9, 8, 9, 8 for Aged EE (solid red), Aged SH (red stripes), Young EE (solid blue), Young SH (blue stripes), respectively). Recognition test of a displaced object 24 hours after training (**B**), shown as a ratio of time spent with the displaced object over all objects, show no difference in group preferences for the displaced object (*F*_3,30_ = 2.216). Test for recognition of a novel object shows differing group preferences for the novel object relative to the pre-existing objects (**C**), shown as a discrimination ratio for the novel object (*F*_3,30_ = 3.316). *p < 0.05, **p < 0.01, ***p < 0.001. Shown as mean ± s.e.m.

We tested another cohort of aged and young mice in a single, displaced and novel object recognition memory (DNOR) task (Fig 3A). Aged and young mice had similar preferences for three pre-determined objects (S5A Fig). 24 hours after training, one object was displaced to an adjacent corner and mice were allowed to explore for 5 minutes. All mice spent equivalent time with all three objects except standard-housed aged mice, which spent more time with familiar object 2 (6.46 ± 1.21 s) than with the displaced object (2.51 ± 0.69 s; p <0.01; S5B Fig). There was no difference in the discrimination ratio between groups (Fig 3B). Following 5 minutes ITI, the mice were then allowed to explore another 5 minutes but with object 2 replaced by a novel object. Aged EE, young EE and young SH mice spent significantly more time with the novel object than with the other two familiar objects, compared to aged SH mice (S5C Fig). Aged EE mice (65.57 ± 7.64%) preferred the novel object 58.5% more than aged SH mice (41.38 ± 6.05%; ANOVA, effect of ‘group’, p < 0.05; Fig 3C). This agrees with previous studies of recognition memory impairment in aged mice [34].

### Protein phosphatases can be manipulated by environmental enrichment

**Fig 4:**
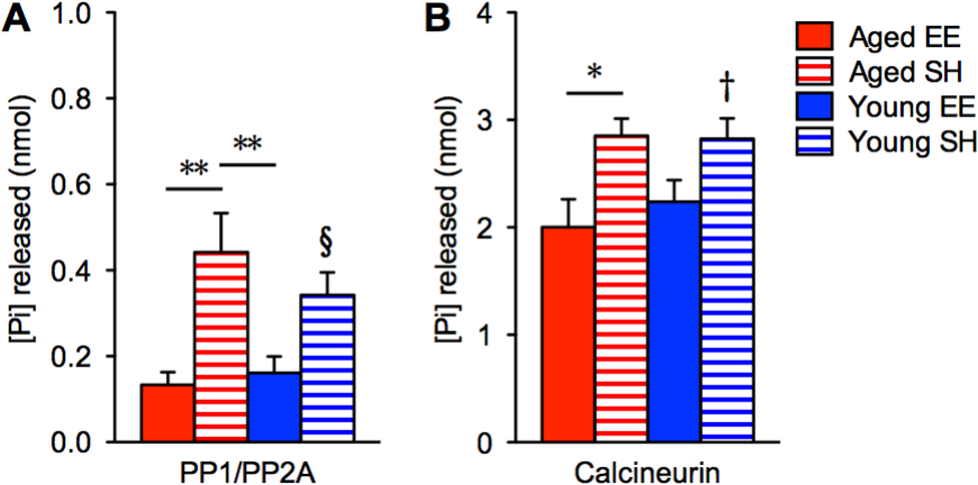
Measure of protein phosphatases’ activity following 6 weeks of respective housing conditions. (**A**) Absolute quantitation of free phosphates released in whole hippocampal fractions due to PP1 and PP2A activities (n = 6, 6, 6, 6 for Aged EE (solid red), Aged SH (red stripes), Young EE (solid blue), Young SH (blue stripes), respectively; *F*_3,20_ = 6.591; ^§^ *p* < 0.05 relative to both aged EE and young EE). (**B**) Free phosphates released due to calcineurin activity (*F*_3,20_ = 4.274; ^†^ *p* < 0.05 relative to aged EE). *p < 0.05, **p < 0.01, ***p < 0.001. Shown as mean ± s.e.m.

We next examined whether the unimpaired cognitive functions in enriched aged mice could be attributed to reductions in PPs. We performed calcineurin and PP1 activity assays in the hippocampus of mice that were tested on DNOR memory tasks following 6 weeks of EE or SH (Fig 3A). Combined PP1 and PP2A activity levels in aged EE (0.13 ± 0.03 nmol) and young EE (0.16 ± 0.04 nmol) mice were significantly lower than in aged SH (0.44 ± 0.09 nmol; p < 0.01) and young SH (0.34 ± 0.05 nmol; p < 0.05) mice (ANOVA, effect of ‘group’, p < 0.01; Fig 4A). Similarly, calcineurin activity was significantly reduced in aged EE (2.00 ± 0.26 nmol) compared to aged SH (2.85 ± 0.16 nmol; p < 0.05) mice, but there was no difference in young (ANOVA, effect of ‘group’, p < 0.05; Fig 4B). This data suggests that EE can reduce dysregulated PPs levels in the brains of aged animals, perhaps as effectively as transgenic inhibition of PPs or even pharmaceutical intervention [14,15].

### Phosphorylated proteins in the hippocampus as an indicator of cognitive health

**Fig 5:**
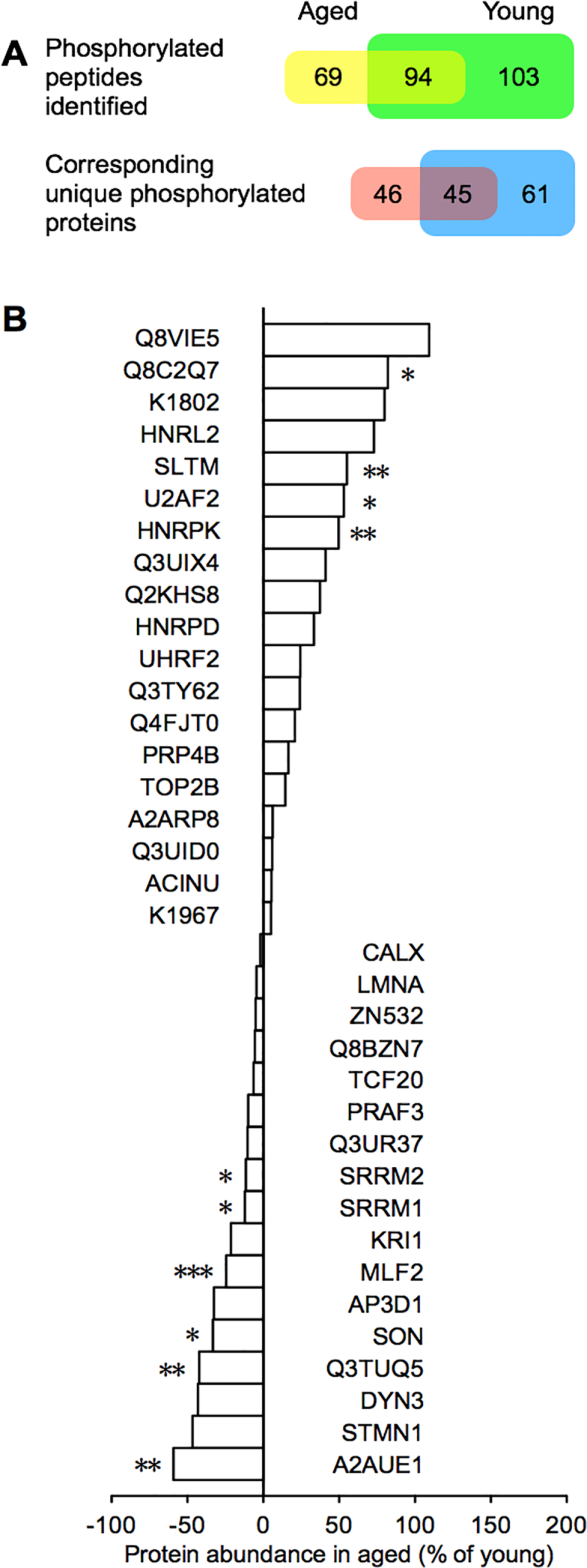
Identification and quantitation of phosphopeptides in naïve aged and young mice using iTRAQ labeling. (**A**) Phosphorylated peptides identified in hippocampal fractions of aged (yellow; n = 9) and young mice (green; n = 15), and the number of phosphopeptides in both aged and young (yellow-green; overlap), using iTRAQ labeling followed by MS/MS analysis. Unique phosphorylated proteins corresponding to the phosphopeptides identified in aged (pink) and young mice (blue), and the number of unique phosphorylated proteins in both aged and young (violet; overlap). (**B**) The relative abundance of phosphorylated proteins in aged compared to young. *p < 0.05, **p < 0.01, ***p < 0.001. Shown as mean ± s.e.m.

To assess the functional implications of increased PP activity with age, we performed iTRAQ quantification of trypsin-digested peptides from hippocampal nuclear protein extracts of naïve aged and naïve young mice. We identified 521 total peptides in aged and 1125 peptides in young with a confidence interval (C.I.) > 95% and significance at p < 0.05 (S1 Table), corresponding to 213 and 328 proteins, respectively. Of those, we identified, in total, 163 phosphorylated peptides (phosphopeptides) in aged and 197 phosphopeptides in young, with 94 phosphopeptides present in both groups (Fig 5A). This corresponded to 91 unique phosphorylated proteins in aged and 106 in young, with 45 present in both groups (Fig 5A). Overall, we identified fewer total peptides, fewer phosphopeptides, and fewer corresponding phosphorylated proteins in aged than in young mice.

We then quantified the relative abundance of phosphopeptides common to both groups. From the 1646 total peptides identified in both aged and young (S1 Table), 233 unique phosphopeptides were identified (S2 Table). Of the 94 phosphopeptides identified in both groups, 67 were found to be unique and we were able to quantify the relative abundance of 56 phosphopeptides in aged relative to young (S3 Table). These 56 unique phosphopeptides correspond to 36 unique proteins present in both aged and young (Fig 5B). We performed gene ontology analysis on the 71 unique phosphopeptides identified in aged but not in young (Table 1 and S4 Table), and on the 95 remaining phosphopeptides identified in young but not in aged (Table 1 and S5 Table). We found aged mice were specifically enriched for phosphorylated proteins and pathways associated with long-term depression, Huntington’s, Parkinson’s, and Alzheimer’s diseases (Tables 1 and 2).

**Table 1:** GO terms for phosphorylated proteins identified in aged but not in young and in young but not in aged.

GO Term	Annotation	Age	Enrichment Ratio*	Adjusted *P*-value
GO:0006397	biological process: mRNA processing	Aged	17.92	6.40E-13
GO:0016071	biological process: mRNA metabolic process	Aged	15.36	2.63E-12
GO:0006396	biological process: RNA processing	Aged	13.52	4.21E-14
GO:0003723	molecular function: RNA binding	Aged	7.37	6.42E-09
GO:0044428	cellular component: nuclear part	Aged	5.62	2.11E-13
GO:0003676	molecular function: nucleic acid binding	Aged	4.84	2.97E-15
GO:0003677	molecular function: DNA binding	Aged	4.08	4.90E-07
GO:0090304	biological process: nucleic acid metabolic process	Aged	3.91	1.69E-13
GO:0005634	cellular component: nucleus	Aged	3.6	1.89E-20
GO:0044446	cellular component: intracellular organelle part	Aged	3.51	3.17E-14
GO:0044422	cellular component: organelle part	Aged	3.42	6.87E-14
GO:0006139	biological process: nucleobase-containing compound metabolic process	Aged	3.41	9.06E-13
GO:0046483	biological process: heterocycle metabolic process	Aged	3.31	1.91E-12
GO:0006725	biological process: cellular aromatic compound metabolic process	Aged	3.3	1.91E-12
GO:0034641	biological process: cellular nitrogen compound metabolic process	Aged	3.21	3.22E-12
GO:1901360	biological process: organic cyclic compound metabolic process	Aged	3.19	3.58E-12
GO:1901363	molecular function: heterocyclic compound binding	Aged	3.13	6.66E-12
GO:0006807	biological process: nitrogen compound metabolic process	Aged	3.11	3.22E-12
GO:0097159	molecular function: organic cyclic compound binding	Aged	3.1	6.66E-12
GO:0000166	molecular function: nucleotide binding	Aged	3.01	8.89E-05
GO:1901265	molecular function: nucleoside phosphate binding	Aged	3.01	8.89E-05
GO:0036094	molecular function: small molecule binding	Aged	2.79	0.0002
GO:0043231	cellular component: intracellular membrane-bounded organelle	Aged	2.45	9.02E-17
GO:0043227	cellular component: membrane-bounded organelle	Aged	2.45	9.02E-17
GO:0043226	cellular component: organelle	Aged	2.25	1.35E-16
GO:0043229	cellular component: intracellular organelle	Aged	2.25	1.35E-16
GO:0044424	cellular component: intracellular part	Aged	2.04	6.89E-16
GO:0005622	cellular component: intracellular	Aged	2.03	1.35E-16
GO:0005515	molecular function: protein binding	Aged	1.85	0.0007
GO:0005488	molecular function: binding	Aged	1.78	2.16E-08
GO:0008380	biological process: RNA splicing	Young	22	1.16E-16
GO:0006397	biological process: mRNA processing	Young	18.16	1.92E-16
GO:0016071	biological process: mRNA metabolic process	Young	15.57	2.28E-15
GO:0006396	biological process: RNA processing	Young	11.38	3.98E-14
GO:0003723	molecular function: RNA binding	Young	7.76	4.04E-12
GO:0044428	cellular component: nuclear part	Young	5.92	4.75E-18
GO:0003676	molecular function: nucleic acid binding	Young	4.66	1.97E-17
GO:0003677	molecular function: DNA binding	Young	4.26	1.92E-09
GO:0032991	cellular component: macromolecular complex	Young	3.93	4.75E-18
GO:0090304	biological process: nucleic acid metabolic process	Young	3.71	4.48E-15
GO:0044446	cellular component: intracellular organelle part	Young	3.47	2.20E-17
GO:0005634	cellular component: nucleus	Young	3.46	3.51E-23
GO:1901363	molecular function: heterocyclic compound binding	Young	3.27	1.57E-16
GO:0006139	biological process: nucleobase-containing compound metabolic process	Young	3.27	1.74E-14
GO:0097159	molecular function: organic cyclic compound binding	Young	3.23	1.69E-16
GO:0046483	biological process: heterocycle metabolic process	Young	3.18	3.98E-14
GO:1901265	molecular function: nucleoside phosphate binding	Young	3.18	1.39E-06
GO:0000166	molecular function: nucleotide binding	Young	3.18	1.39E-06
GO:0006725	biological process: cellular aromatic compound metabolic process	Young	3.17	4.08E-14
GO:1901360	biological process: organic cyclic compound metabolic process	Young	3.13	3.48E-14
GO:0036094	molecular function: small molecule binding	Young	2.95	5.30E-06
GO:0044260	biological process: cellular macromolecule metabolic process	Young	2.84	1.74E-14
GO:0005515	molecular function: protein binding	Young	2.37	1.39E-10
GO:0043231	cellular component: intracellular membrane-bounded organelle	Young	2.31	2.19E-17
GO:0043227	cellular component: membrane-bounded organelle	Young	2.3	2.20E-17
GO:0043226	cellular component: organelle	Young	2.17	7.60E-18
GO:0043229	cellular component: intracellular organelle	Young	2.17	7.60E-18
GO:0044424	cellular component: intracellular part	Young	1.98	2.19E-17
GO:0005622	cellular component: intracellular	Young	1.96	8.78E-18
GO:0005488	molecular function: binding	Young	1.91	3.71E-14

* Sorted according to treatment followed by enrichment ratio. P-values adjusted for multiple comparisons using Benjamini-Hochberg correction.

**Table 2:** KEGG pathways of phosphorylated proteins identified in aged but not in young and in young but not in aged.

Pathway	Age	Enrichment Ratio	Adjusted *P*-value*
mRNA surveillance pathway	Aged	57.43	2.13E-08
Spliceosome	Aged	32.25	5.02E-06
RNA transport	Aged	26.49	8.80E-06
Fc gamma R-mediated phagocytosis	Aged	29.67	0.0005
Huntington's disease	Aged	13.56	0.0057
Gastric acid secretion	Aged	24.39	0.0066
Salivary secretion	Aged	23.12	0.0066
Long-term depression	Aged	24.73	0.0066
Phosphatidylinositol signaling system	Aged	22.82	0.0066
Long-term potentiation	Aged	25.8	0.0066
Gap junction	Aged	20.23	0.0078
GnRH signaling pathway	Aged	17.98	0.0089
Pancreatic secretion	Aged	17.12	0.0091
Vascular smooth muscle contraction	Aged	14.47	0.0115
Parkinson's disease	Aged	12.03	0.0155
Calcium signaling pathway	Aged	10	0.0204
Alzheimer's disease	Aged	9.47	0.0213
MAPK signaling pathway	Aged	6.64	0.0387
Spliceosome	Young	44.92	1.03E-11
RNA transport	Young	20.5	4.24E-05
mRNA surveillance pathway	Young	29.63	6.18E-05
Systemic lupus erythematosus	Young	18.49	0.0003
GnRH signaling pathway	Young	20.87	0.0014
Calcium signaling pathway	Young	11.61	0.0065
Salivary secretion	Young	17.89	0.0108
Long-term potentiation	Young	19.97	0.0108
Gastric acid secretion	Young	18.87	0.0108
Pancreatic secretion	Young	13.25	0.0172
Oocyte meiosis	Young	12.19	0.0184
Vascular smooth muscle contraction	Young	11.2	0.0197
Protein processing in endoplasmic reticulum	Young	8.15	0.033
Phagosome	Young	7.83	0.033
Chemokine signaling pathway	Young	7.45	0.0339
Huntington's disease	Young	6.99	0.0356
Endocytosis	Young	6.26	0.0409

* Sorted according to treatment followed by P-value, using Benjamini-Hochberg correction for multiple comparisons.

Additionally, we performed DAVID analysis of the phosphorylated proteins identified in aged (S6 Table), in aged but not in young (S7 Table), in young (S8 Table), in young but not in aged (S9 Table), and in both aged and young (S10 Table). In aged mice, we found enriched biological processes associated with negative regulation of gene expression, supporting the evidence that aged mice have globally reduced gene expression levels. In contrast, we found positive processes associated with neuronal activity and synaptic plasticity enriched in the hippocampus of young mice.

### The effect of age and environment on the hippocampal transcriptome

Aging is known to down-regulate transcriptional networks [23–25,32], however, learning and environmental enrichment has been shown to increase gene expression [45,46]. To determine the effects of age and environment on transcriptional activity in the brain, we used SAGE-Seq to analyze hippocampal transcriptome profiles.

**Fig 6:**
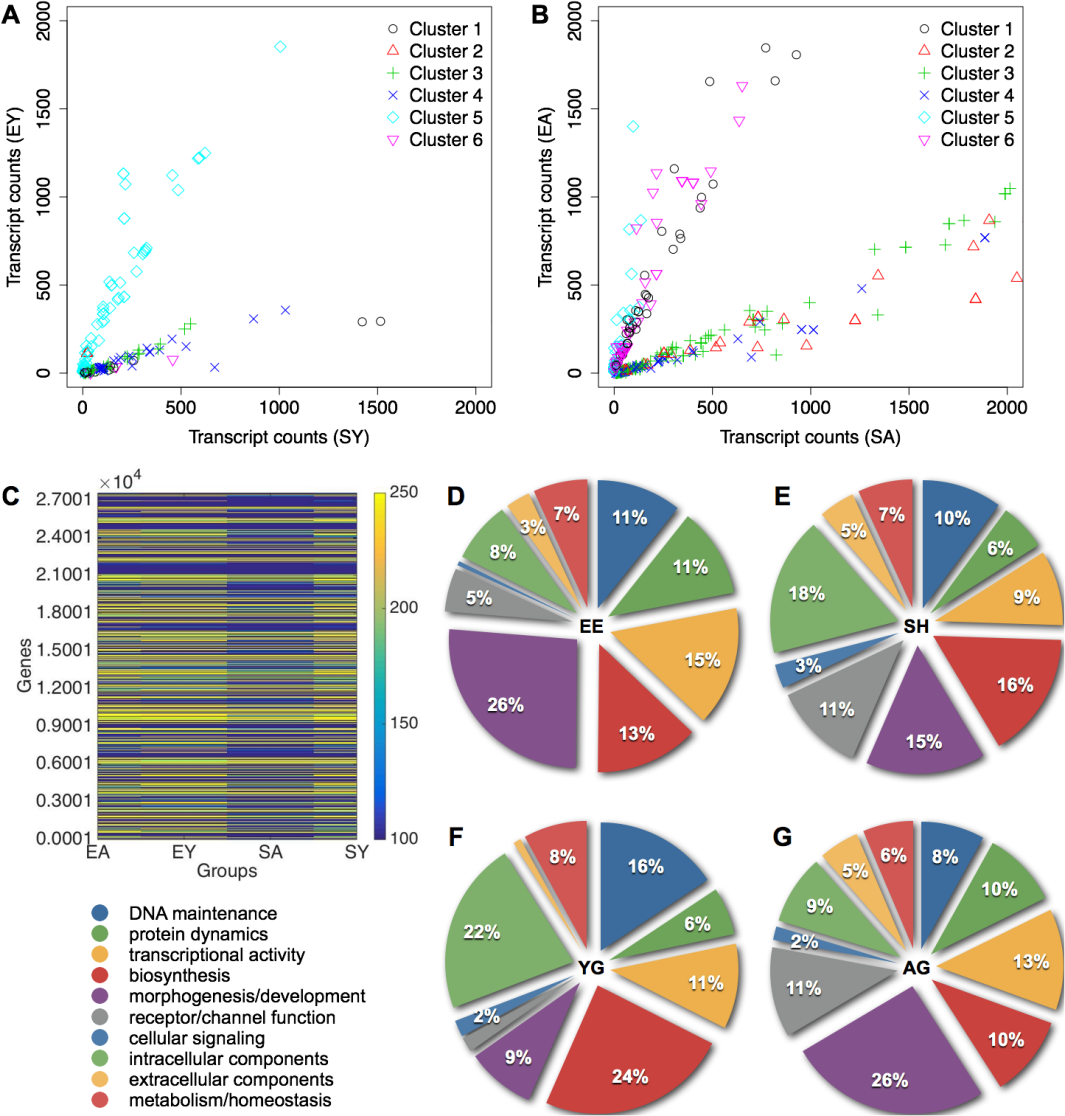
Hippocampal genes associated with EE in aged and young mice using SAGE-Seq. (**A**) Unbiased clustering of transcripts differentially expressed in young EE (EY) compared to young SH (SY). Clusters 2 and 5 are associated with EY and clusters 1, 3, 4, and 6 are associated with SY. Counts are cutoff at 2000 for visualization. (**B**) Clustering of transcripts differentially expressed in aged EE (EA) compared to aged SH (SA). Clusters 1, 5, and 6 are associated with EA and clusters 2, 3, and 4 are associated with SA. (**C**) Heatmap of the 27,581 genes shown as absolute expression levels greater than 250 transcripts (yellow), less than 100 transcripts (blue), and counts inbetween are in shades of green. Transcript levels range from 0–76,000, and EA average of 513 and median of 37, EY average of 484 and median of 42, SA average of 285 and median of 21, and SY average of 455 and median of 40. Distribution of GO categories in (**D**) EE, (**E**) SH, (**F**) young (YG), and (**G**) aged (AG). GO terms were classified into ten functional groups including, clockwise from DNA maintenance (blue), protein dynamics (green), transcriptional activity (orange), biosynthesis (red), morphogenesis and developmental processes (violet), receptor and channel function (grey), cellular signaling pathways (cornflower blue), intracellular components (pale green), extracellular components (pale orange), and metabolic and homeostatic functions (pale red). The broader categories of binding, cell, and metabolism were excluded.

Comparative counts identified clusters 2 and 5 enriched in young EE (EY) and clusters 1, 3, 4, and 6 enriched in young SH (SY) (Fig 6A; S11 and S12 Tables). In aged, clusters 1, 5, and 6 were enriched in aged EE (EA) and clusters 2, 3, and 4 were enriched in aged SH (SA) (Fig 6B; S13 and S14 Tables). We identified 59 genes differentially expressed in young, and 410 genes differentially expressed in aged using edgeR at an FDR of 10%, a stringent method for multi-group comparisons that utilizes empirical Bayes estimation and exact tests based on the negative binomial distribution (refer to Methods). This suggests that EE altered a considerable number of genes between aged but not between young mice. Aged SH mice had generally lower levels of gene expression across the 27,851 gene transcripts compared to aged EE, young EE, and young SH mice (Fig 6C, S6A–S6L Fig).

We also found a 3 to 6-fold increase in the expression of *Ppp1r1a*, *Ppp1r16b*, *Ppp1r3b*, and *Ppp1r13b* in aged EE compared to aged SH mice, transcripts that correspond to regulatory inhibitor subunits of PP1 (S13 and S14 Tables). In a separate study, another regulatory inhibitor subunit, *Ppp1r14a* expression, was found to be increased in aged-unimpaired mice, which have limited cognitive deficits similar to aged EE mice [23]. Furthermore, in agreement with the higher PP1/PP2A activity we observed in aged SH mice (Fig 4A), other microarray studies similarly show that expression of regulatory subunits of PP2A, such as *Ppp2r5a* and *Ppp2r2d*, are increased in aging [24] but decreased in aged-unimpaired mice [23], respectively.

We performed DAVID analysis on differentially expressed transcripts with a Benjamini and Hochberg-adjusted p < 0.01 to obtain functional categories enriched in the respective groups (S15–S18 Tables). Annotated GO terms were classified into higher-order GO slim ancestral terms to identify relevant functional groups associated with age and housing (Fig 6D–6G). With EE, categories associated with protein dynamics, transcriptional activity, and morphogenetic and developmental processes had higher representations at the expense of receptor/channel function and intracellular components enriched in SH (Fig 6D and 6E). In young mice, DNA maintenance, biosynthesis, and intracellular components were higher over those of morphogenetic and developmental processes, and receptor/channel function enriched in aged (Fig 6F and 6G).

We found similarities to DAVID analysis when analyzing individual GO terms. In young mice, neuronal and synaptic transmission was associated with EE while oxidative stress, DNA hypermethylation (a process to silence genes) and cell death were associated with SH (S19 and S20 Tables). In aged mice, EE was significantly enriched for gene transcription-dependent processes suggesting increased transcriptional activity with EE (S21 and S22 Tables). Using KEGG pathway analysis, we also found that transcriptional regulation-associated pathways were enriched in young EE, while Huntington’s, Parkinson’s, and Alzheimer’s disease pathways were enriched in young SH (S23 and S24 Tables). In aged mice, EE was associated with memory-specific pathways including MAPK signaling, axon guidance, and long-term potentiation, while SH was associated with neurodegenerative disease pathways including amyotrophic lateral sclerosis and Alzheimer’s disease, comparable to those found in young SH mice (S25 and S26 Tables). Neurodegenerative pathways identified in the transcriptome profiles of young SH and aged SH, which together constitute the SH condition, are nearly identical to those identified in the proteomes of naïve aged mice (Tables 1 and 2).

## Discussion

Our study provides a comprehensive investigation of the behavioral, cellular, and transcriptional processes associated with the progression of ARCD as well as the preservation of cognitive function with EE. More specifically, it reveals that dysregulation of PPs in the hippocampus may underlie ARCD, but that EE-mediated increase in PP1 inhibitors during middle-age can preserve cognitive function. This study also provides in-depth analysis of the aging proteome and transcriptome in the hippocampus. Transcriptome analysis reveals networks and pathways associated with cellular senescence and neural functions but also to novel processes not yet investigated in the context of aging and ARCD. Importantly, pathway analysis shows that neurodegenerative processes are ameliorated in enriched aged mice. Proteomic analysis provides further evidence that phosphorylation is dysregulated in aging and provides novel targets for intervention in ARCD and neurodegenerative diseases.

Using massively parallel sequencing, which allows for absolute rather than relative quantification and greater, deeper coverage of the genome, we procured a unique dataset with higher confidence levels than previously possible with similarly powered microarray analysis. We applied the GLM functionality in the well-established edgeR package to our dataset [39,47,48]. This is a stringent method that uses Benjamini-Hochberg procedure by default to obtain p values adjusted for multiple testing rather than other methods, such as Storey-Tibshirani, which can increase the number of significant genes by more than 30% at the same FDR [49–52]. Moreover, normalization methods, including edgeR, have also been shown to greatly underestimate the number of truly positive differentially expressed genes by up to half [49,50,52,53]. Underestimation in addition to less stringent methods will result in larger gene lists at the same FDR value or lower FDR rates for the same distribution. Thus, our list of genes are likely to be underrepresented, but for the purposes of extracting a focused set of differentially expressed genes, specifically those belonging to the PPs family, we deemed edgeR a suitable choice for comparative analysis.

Aged animals are known to have globally decreased gene expression levels [23–28,32,34,35]. We therefore expected greater variance in gene expression levels in aged mice due to increased variance with age, disease and environmental enrichment. The global assessment of gene expression changes reflects this difference, with aged SH mice having considerably lower levels of gene expression across the 27,851 gene transcripts compared to aged EE, young EE, and young SH mice (Fig 6C). This suggests the cognitive improvements that have accompanied aged EE mice are likely due to the effect of environmental enrichment and accompanying changes to the transcriptome. Increased variance with aging may also explain why young mice had relatively few differentially expressed genes–the hippocampi and cognitive functions of young mice are equally healthy and plastic regardless of enrichment.

In general, there are relatively few genome-wide datasets in the brain, particularly using next generation sequencing techniques, and to our knowledge we are not aware of any genome-wide studies detailing age-related cognitive impairment and its rescue or preservation through environmental enrichment. There are, however, several microarray-based gene expression studies investigating similar age-related phenomena, specifically relating to memory and aging [23,24,34,54]. These studies of hippocampal gene expression in aged mice have identified similar neurodegenerative disease pathways in aging and support our finding that PP1/PP2A activity and expression is increased in aged mice [23,24,32,34,54]. However, our study shows that the mechanism for the progression of ARCD and its prevention through EE may be attributed, in particular, to the regulation of PP1 inhibitors–including *Ppp1r1a*, *Ppp1r3b*, *Ppp1r13b*, *Ppp1r14a*, and *Ppp1r16b* found in our study and others.

PP1 has also been implicated in the regulation of histone phosphorylation and acetylation [38,55,56], and has been shown to regulate chromosome condensation and segregation [57–59]. The importance of epigenetic processes in the brain is highlighted by recent studies that show chromatin remodeling is also dysregulated in aging. Like PPs, histone deacetylase (HDAC) inhibitors have been shown to enhance or reverse memory deficits associated with aging and neurodegeneration [7,37,60]. However, the functional activities of HDACs as well as the phosphorylation and acetylation states of histones are likely regulated by the activities of protein kinases and protein phosphatases such as PP1 [38,55,56,61]. It may, therefore, be possible to conclude that dysregulated epigenetic processes in aging are linked to dysregulated PPs and the associated gene expression changes in the aging brain.

## Conclusions

Further studies are needed to understand the cellular and genetic processes involved in cognitive aging, however, this study provides an in-depth overview of the genes and pathways affected in ARCD. In particular, the discovery of PP dysregulation in aging as a process that can be manipulated by behavioral modification provides both a novel strategy with which to prevent ARCD, but also a novel class of targets for pharmacological intervention. Our emphasis on preservation of cognitive functions during middle-age using environmental and behavioral modifications, provides a sensible approach to address the aging population and the financial and social burdens facing society.

## Supporting Information

S1 Fig Standard laboratory housing and environmental enrichment cages with enrichment variables and their arrangements. (**A**) Standardized laboratory cages with a cardboard carton for housing and tissues for nesting materials used in the animal facilities at the University of Zürich. (**B**) Environmental enrichment cages adapted from Tecniplast cages for rats. The size of the enrichment cage in the photo is approximate to standard laboratory cages (refer to Methods for dimensions). (**C**) The configuration of enrichment materials, shown in four different example arrangements, were alternated on a weekly basis. (**D**) The enrichment materials–toys, mazes, tunnels, housing, and running wheels–used for each cage over the course of the enrichment experiments.
(TIF)

S2 Fig 2-Context fear conditioning apparatus. (A) The clear, acrylic conditioning container and electric grid included in the TSE Fear Conditioning Systems was used as the first Context. (B) An infra-red light-penetrable, black acrylic conditioning container was used in conjunction with a custom-designed electric grid consisting of 1.25 cm metal strips with regularly spaced drilled holes measuring 0.5 cm in diameter, as the second Context.
(TIF)

S3 Fig Weight and open field behavior before and after enrichment or standard housing. (**A**) Weight of aged and young mice followed by (**B**) open field test measuring time spent in the center of the box from the fear conditioning cohort before separation into respective housing cages. (**C**) Weight of mice by age after several weeks of enrichment or standard housing and (**D**) separated by age, before and after (^†^ *p* < 0.001 relative to both Aged EE and Aged SH). (**E**) Weight of mice separated by age and EE or SH (Aged EE (solid red), Aged SH (red stripes), Young EE (solid blue), Young SH (blue stripes); ^§^ *p* < 0.001 relative to Aged SH only). (**F**) Time spent in center in the open field test separated by age and housing after EE or SH. *p < 0.05, **p < 0.01, ***p < 0.001. Shown as mean ± s.e.m.
(TIF)

S4 Fig Swim speed and platform crossings of aged and young mice in the Morris water maze before and after enrichment or standard housing. (**A**) The average swim speed of aged and young mice to acquire the hidden platform across 10 days of training in MWM1. (**B**) The average swim speed of mice during the probe trial on day 11 with the hidden platform removed and a trial time of 60 seconds. (**C**) The average number of platform crossings by quadrant during the probe trial on day 11 (T, target; R, right; L, left; O, opposite). (**D**) The average swim speed of aged and young mice during the probe trial on day 10 with the hidden platform removed and duration of 60 seconds. (**E**) The average swim speed of mice separated by EE and SH during the probe trial (Aged EE (solid red), Aged SH (red stripes), Young EE (solid blue), Young SH (blue stripes); ^§^ *p* < 0.05 relative to both Aged EE and Aged SH). (**F**) The average number of platform crossings by quadrant during the probe trial on day 10. (**G**) Average platform crossings in mice separated by EE and SH during the probe trial. *p < 0.05, **p < 0.01, ***p < 0.001. Shown as mean ± s.e.m.
(TIF)

S5 Fig Object characterization and preference in the DNOR task and changes in weight before and after enrichment or standard housing. (**A**) Average time spent for each of the three objects from day 1 trial 1 as a measure of pre-training exploration times and object preference by each group. Recognition test of a displaced object 24 hours after training shown (**B**) by object (*p* values relative to the displaced object), and test for recognition of a novel object relative to pre-existing objects shown (**C**) by object (*p* values relative to the novel object). Weight of mice (**D**) at the onset of behavioral testing and (**E**) after testing followed by several weeks of housing. (**F**) Changes in weight by age, before and after EE or SH (^†^ *p* < 0.001 relative to both Aged EE and Aged SH). (**G**) Weight of mice separated by age and EE or SH (Aged EE (solid red), Aged SH (red stripes), Young EE (solid blue), Young SH (blue stripes); ^§^ *p* < 0.001 relative to Aged SH only). *p < 0.05, **p < 0.01, ***p < 0.001. Shown as mean ± s.e.m.
(TIF)

S6 Fig Comparative scatter and volcano plots of significantly expressed genes. Scatter and volcano plots of genes significantly expressed in (**A, B**) aged EE (EA) versus young EE (EY), (**C, D**) EY versus aged SH (SA), (**E, F**) EA versus SA, (**G, H**) EY versus young SH (SY), (**I, J**) SY versus EA, and (**K, L**) SY versus SA. Scatter plots are shown as absolute expression levels based on comparative counts using the general linearized model (GLM) method in edgeR and volcano plots are shown as-log10(FDR) versus log2 ratio of the respective pairings.
(TIF)

S1 Table All peptides identified in aged and young. (XLSX)

S2 Table Unique phosphopeptides identified in total. (XLSX)

S3 Table Unique phosphopeptides identified in both aged and young. (XLSX)

S4 Table Unique phosphopeptides identified in aged but not in young. (XLSX)

S5 Table Unique phosphopeptides identified in young but not in aged. (XLSX)

S6 Table Functional annotation of unique phosphorylated proteins in aged. (XLSX)

S7 Table Functional annotation of unique phosphorylated proteins in aged but not in young. (XLSX)

S8 Table Functional annotation of unique phosphorylated proteins in young. (XLSX)

S9 Table Functional annotation of unique phosphorylated proteins in young but not in aged. (XLSX)

S10 Table Functional annotation of unique phosphorylated proteins in both aged and young. (XLSX)

S11 Table Differentially expressed genes in young EE over young SH. (XLSX)

S12 Table Differentially expressed genes in young SH over young EE. (XLSX)

S13 Table Differentially expressed genes in aged EE over aged SH. (XLSX)

S14 Table Differentially expressed genes in aged SH over aged EE. (XLSX)

S15 Table Functional annotation of differentially expressed genes in young EE over young SH. (XLSX)

S16 Table Functional annotation of differentially expressed genes in young SH over young EE. (XLSX)

S17 Table Functional annotation of differentially expressed genes in aged EE over aged SH. (XLSX)

S18 Table Functional annotation of differentially expressed genes in aged SH over aged EE. (XLSX)

S19 Table Gene ontology analysis of differentially expressed genes in young EE over young SH. (XLSX)

S20 Table Gene ontology analysis of differentially expressed genes in young SH over young EE. (XLSX)

S21 Table Gene ontology analysis of differentially expressed genes in aged EE over aged SH. (XLSX)

S22 Table Gene ontology analysis of differentially expressed genes in aged SH over aged EE. (XLSX)

S23 Table KEGG pathway analysis of differentially expressed genes in young EE over young SH. (XLSX)

S24 Table KEGG pathway analysis of differentially expressed genes in young SH over young EE. (XLSX)

S25 Table KEGG pathway analysis of differentially expressed genes in aged EE over aged SH. (XLSX)

S26 Table KEGG pathway analysis of differentially expressed genes in aged SH over aged EE. (XLSX)
