# Supplementary figures and images for: Integrative Transcriptome Profiling of Cognitive Aging and Its Preservation through Ser/Thr Protein Phosphatase Regulation
Source: PLoS One. 2015 Jun 23;10(6):e0130891. doi: 10.1371/journal.pone.0130891 (PMC4478024; doi:10.1371/journal.pone.0130891)

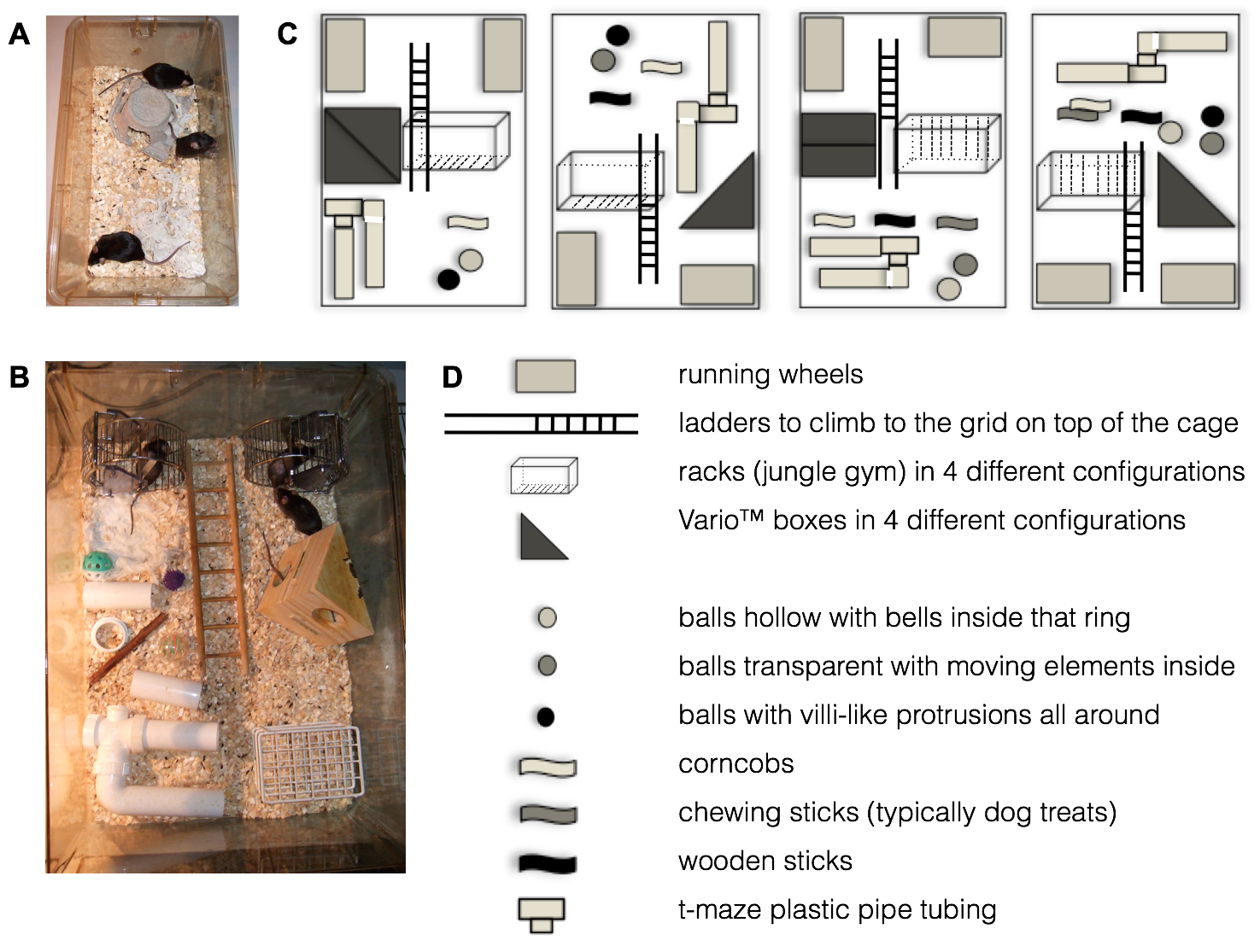

Supplement: S1 Fig — (A) Standardized laboratory cages with a cardboard carton for housing and tissues for nesting materials used in the animal facilities at the University of Zürich. (B) Environmental enrichment cages adapted from Tecniplast cages for rats. The size of the enrichment cage in the photo is approximate to standard laboratory cages (refer to Methods for dimensions). (C) The configuration of enrichment materials, shown in four different example arrangements, were alternated on a weekly basis. (D) The enrichment materials–toys, mazes, tunnels, housing, and running wheels–used for each cage over the course of the enrichment experiments. (TIF) [file pone.0130891.s001.tif]

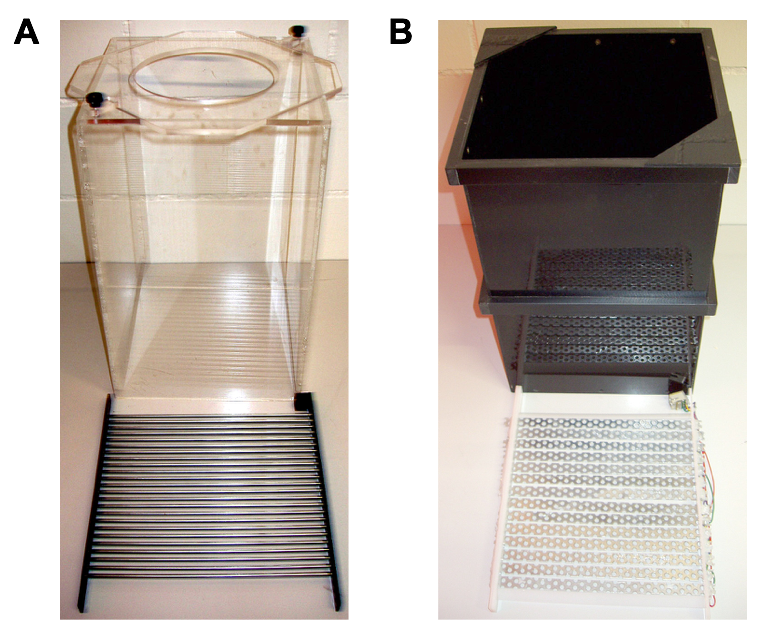

Supplement: S2 Fig — (A) The clear, acrylic conditioning container and electric grid included in the TSE Fear Conditioning Systems was used as the first Context. (B) An infra-red light-penetrable, black acrylic conditioning container was used in conjunction with a custom-designed electric grid consisting of 1.25 cm metal strips with regularly spaced drilled holes measuring 0.5 cm in diameter, as the second Context. (TIF) [file pone.0130891.s002.tif]

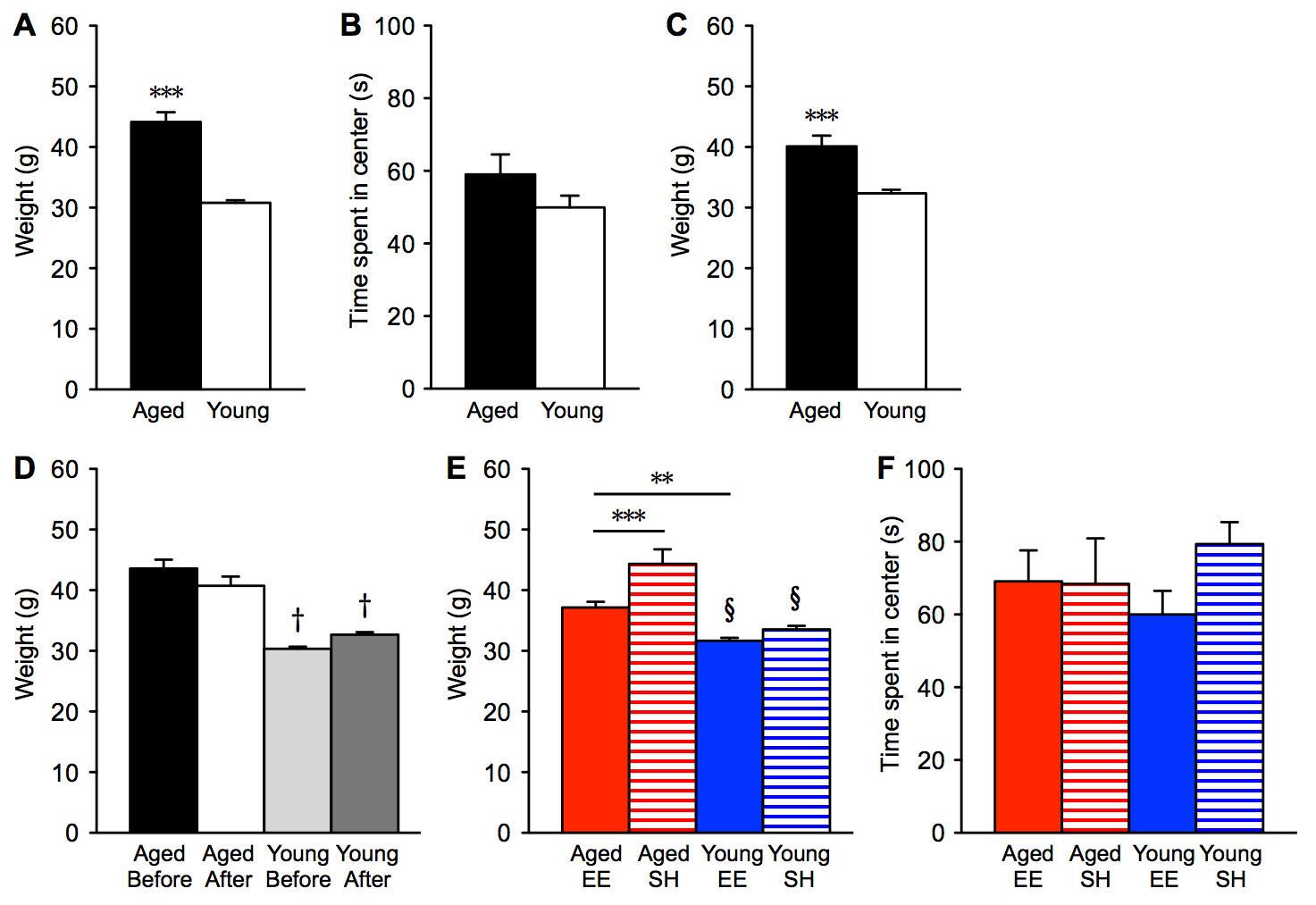

Supplement: S3 Fig — (A) Weight of aged and young mice followed by (B) open field test measuring time spent in the center of the box from the fear conditioning cohort before separation into respective housing cages. (C) Weight of mice by age after several weeks of enrichment or standard housing and (D) separated by age, before and after († p < 0.001 relative to both Aged EE and Aged SH). (E) Weight of mice separated by age and EE or SH (Aged EE (solid red), Aged SH (red stripes), Young EE (solid blue), Young SH (blue stripes); § p < 0.001 relative to Aged SH only). (F) Time spent in center in the open field test separated by age and housing after EE or SH. *p < 0.05, **p < 0.01, ***p < 0.001. Shown as mean ± s.e.m. (TIF) [file pone.0130891.s003.tif]

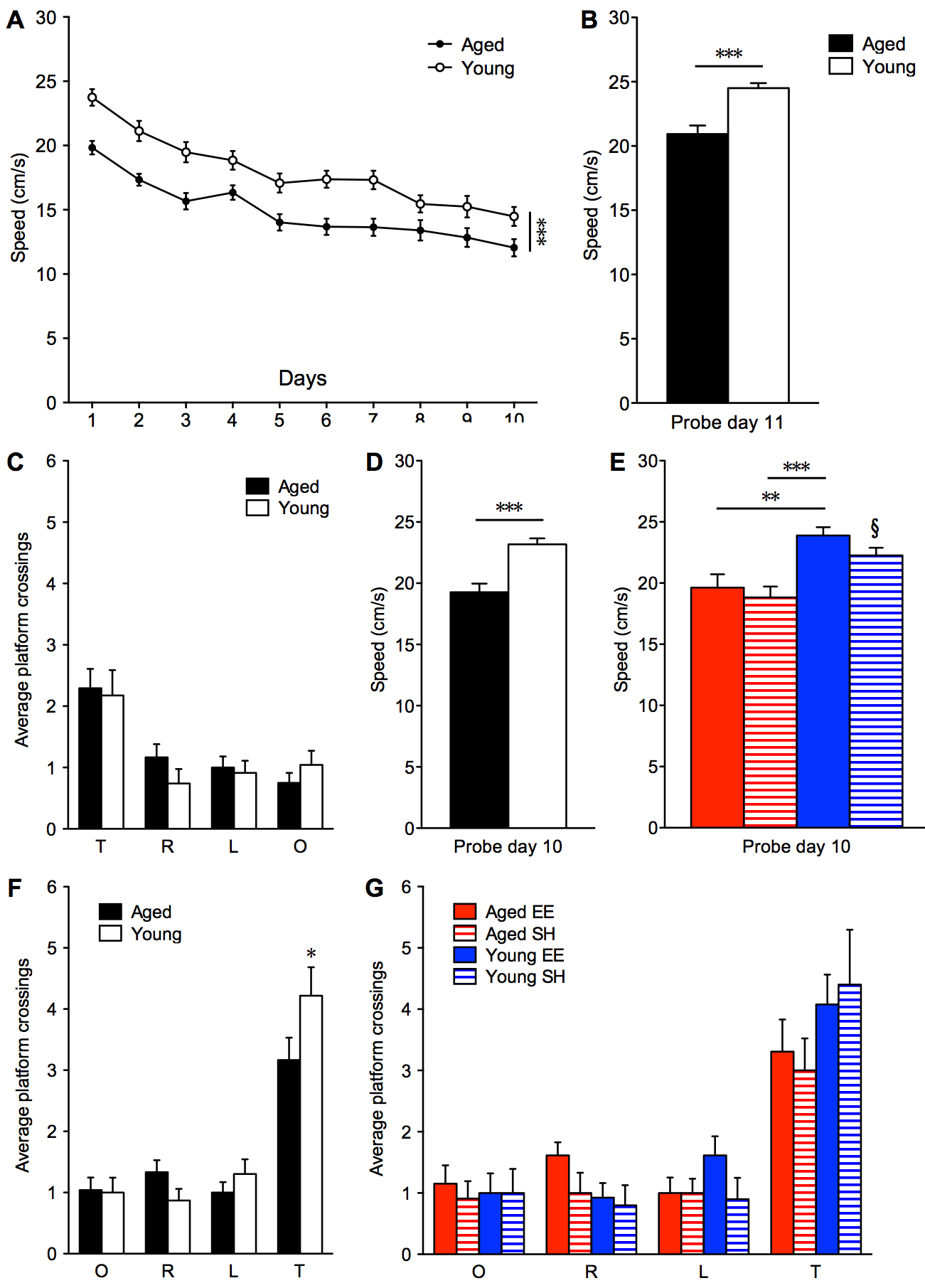

Supplement: S4 Fig — (A) The average swim speed of aged and young mice to acquire the hidden platform across 10 days of training in MWM1. (B) The average swim speed of mice during the probe trial on day 11 with the hidden platform removed and a trial time of 60 seconds. (C) The average number of platform crossings by quadrant during the probe trial on day 11 (T, target; R, right; L, left; O, opposite). (D) The average swim speed of aged and young mice during the probe trial on day 10 with the hidden platform removed and duration of 60 seconds. (E) The average swim speed of mice separated by EE and SH during the probe trial (Aged EE (solid red), Aged SH (red stripes), Young EE (solid blue), Young SH (blue stripes); § p < 0.05 relative to both Aged EE and Aged SH). (F) The average number of platform crossings by quadrant during the probe trial on day 10. (G) Average platform crossings in mice separated by EE and SH during the probe trial. *p < 0.05, **p < 0.01, ***p < 0.001. Shown as mean ± s.e.m. (TIF) [file pone.0130891.s004.tif]

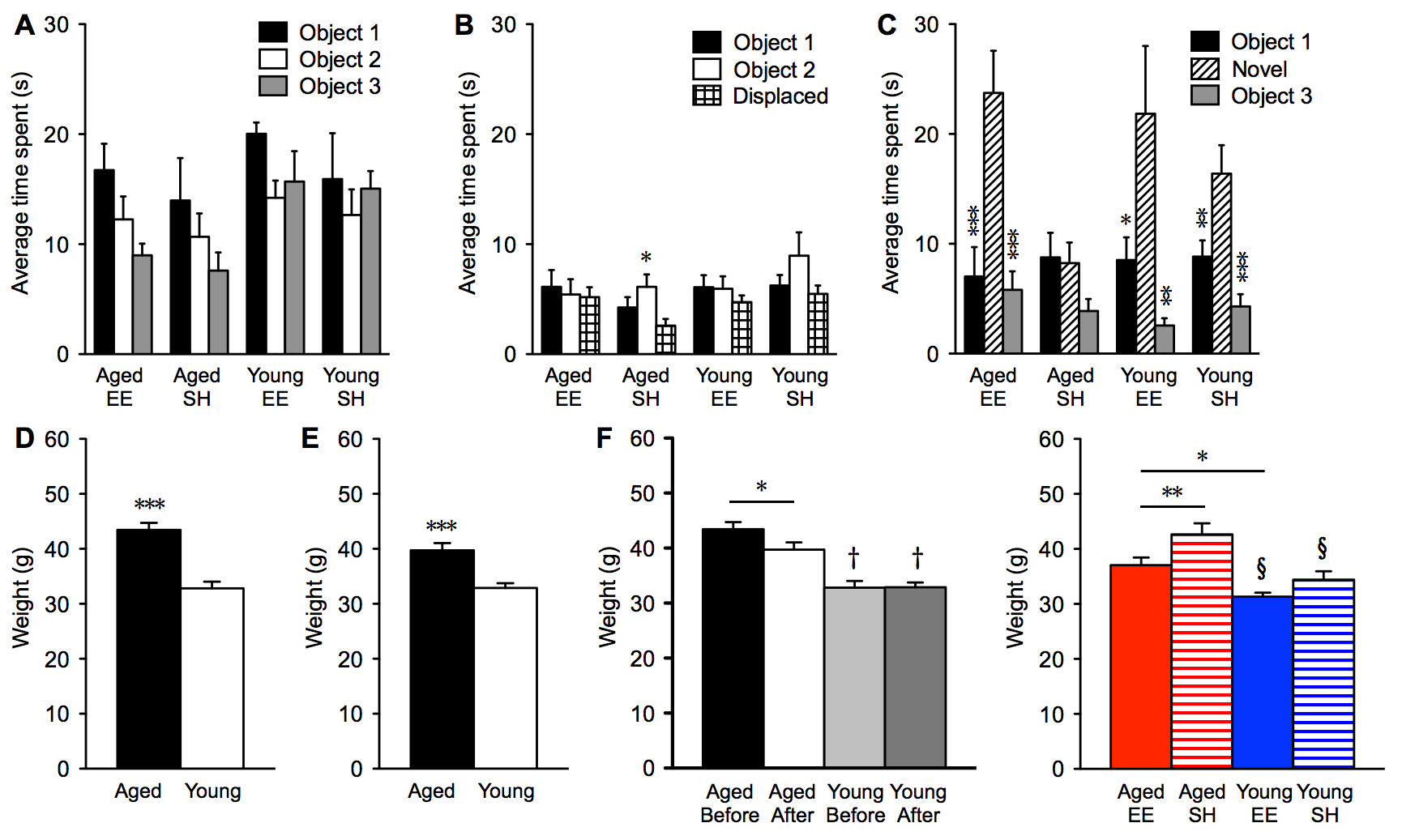

Supplement: S5 Fig — (A) Average time spent for each of the three objects from day 1 trial 1 as a measure of pre-training exploration times and object preference by each group. Recognition test of a displaced object 24 hours after training shown (B) by object (p values relative to the displaced object), and test for recognition of a novel object relative to pre-existing objects shown (C) by object (p values relative to the novel object). Weight of mice (D) at the onset of behavioral testing and (E) after testing followed by several weeks of housing. (F) Changes in weight by age, before and after EE or SH († p < 0.001 relative to both Aged EE and Aged SH). (G) Weight of mice separated by age and EE or SH (Aged EE (solid red), Aged SH (red stripes), Young EE (solid blue), Young SH (blue stripes); § p < 0.001 relative to Aged SH only). *p < 0.05, **p < 0.01, ***p < 0.001. Shown as mean ± s.e.m. (TIF) [file pone.0130891.s005.tif]

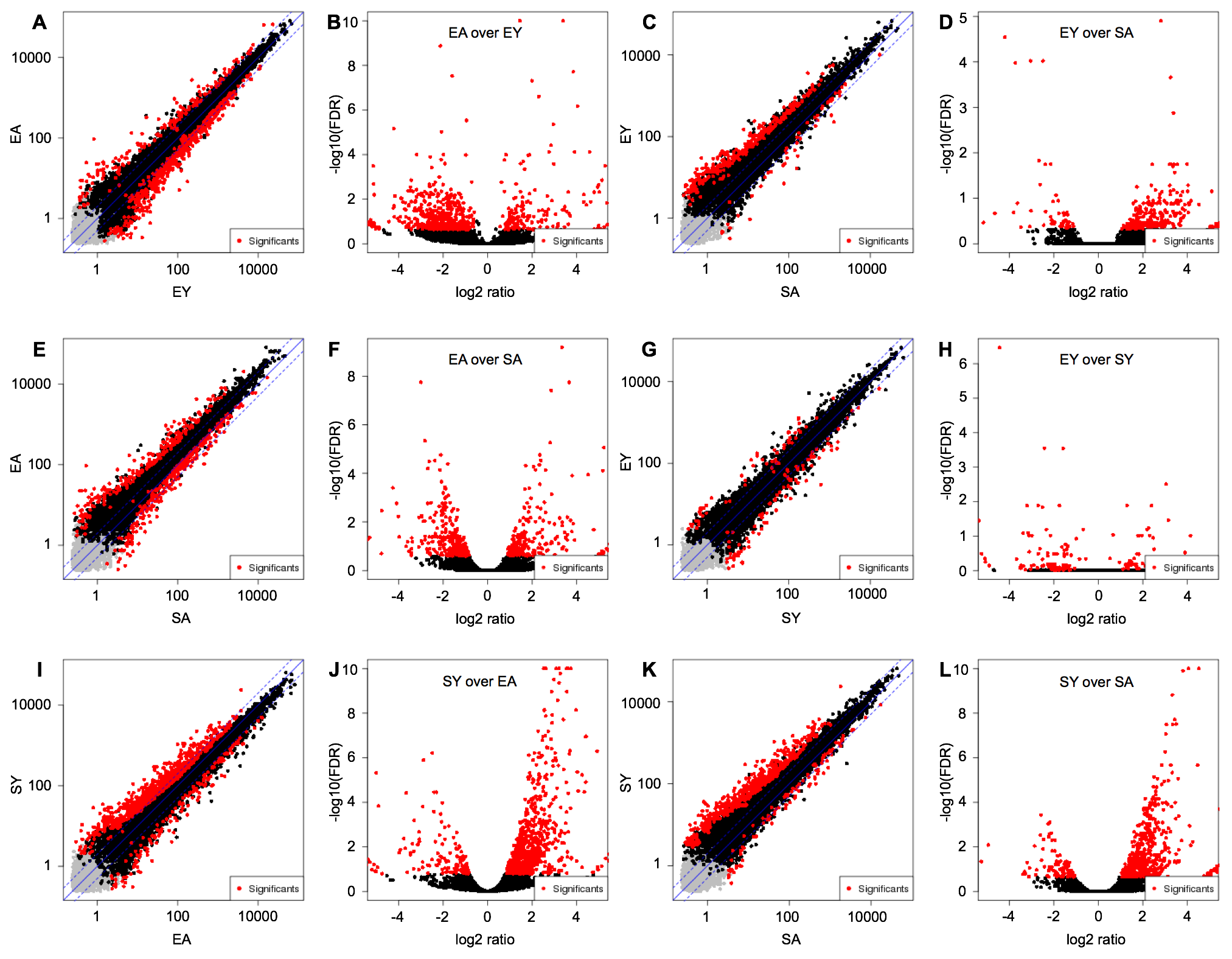

Supplement: S6 Fig — Scatter and volcano plots of genes significantly expressed in (A, B) aged EE (EA) versus young EE (EY), (C, D) EY versus aged SH (SA), (E, F) EA versus SA, (G, H) EY versus young SH (SY), (I, J) SY versus EA, and (K, L) SY versus SA. Scatter plots are shown as absolute expression levels based on comparative counts using the general linearized model (GLM) method in edgeR and volcano plots are shown as-log10(FDR) versus log2 ratio of the respective pairings. (TIF) [file pone.0130891.s006.tif]
